# Transcriptional Network of p63 in Human Keratinocytes

**DOI:** 10.1371/journal.pone.0005008

**Published:** 2009-03-25

**Authors:** Silvia Pozzi, Federico Zambelli, Daniele Merico, Giulio Pavesi, Amélie Robert, Peggy Maltère, Xavier Gidrol, Roberto Mantovani, M. Alessandra Vigano

**Affiliations:** 1 Department of Biomolecular Sciences and Biotechnologies, University of Milan, Milan, Italy; 2 CEA, Laboratoire d'Exploration Fonctionnelle des Génomes, Evry, France; Institute of Genetics and Molecular and Cellular Biology, France

## Abstract

p63 is a transcription factor required for the development and maintenance of ectodermal tissues in general, and skin keratinocytes in particular. The identification of its target genes is fundamental for understanding the complex network of gene regulation governing the development of epithelia. We report a list of almost 1000 targets derived from ChIP on chip analysis on two platforms; all genes analyzed changed in expression during differentiation of human keratinocytes. Functional annotation highlighted unexpected GO terms enrichments and confirmed that genes involved in transcriptional regulation are the most significant. A detailed analysis of these transcriptional regulators in condition of perturbed p63 levels confirmed the role of p63 in the regulatory network. Rather than a rigid master-slave hierarchical model, our data indicate that p63 connects different hubs involved in the multiple specific functions of the skin.

## Introduction

p63 is a transcription factor homologous to p53 and p73 [1] which binds DNA in a sequence-specific way. p63 has two different transcription initiation sites generating proteins containing (TA) or lacking (ΔN) an activation domain. The 3′ end of the gene is involved in alternative splicing of three isoforms termed α, β and γ. Hence, a minimum of six p63 isoforms are present in cells, at various levels of relative expression. Unlike p53, p63 and p73 are not ubiquitously expressed, and are involved in developmental processes. In particular, compelling genetic evidence in mouse [2], [3], human [4] and zebrafish [5], [6] indicates that p63 plays a central role in development of ectodermal tissues. It is clear therefore, that p63 is a master regulatory gene of multi-layered epithelia in general, and of keratinocytes in particular [7].

Identification of targets is crucial in order to understand the developmental strategy sustained by p63. Several approaches have been taken over the last few years, notably expression profiles of p63 overexpressing or p63 silenced cells [8] and genome wide p63 ChIP on chip analysis [9], [10]. Most of these studies provided good evidences that p63 governs specific programs involved in epithelial differentiation such as adhesion [11] or cancer progression [12]. We undertook a ChIP on chip screening with the human keratinocyte cell line HaCaT, which predominantly expresses the ΔNp63α isoform and identified 186 high confidence p63 targets, which were validated in different biological assays [10]. We reanalyzed these binding data with less stringent criteria and extended the list of targets to over 1000 and confirm the pivotal role of p63 in transcriptional regulation. An extensive validation of targets involved in transcription in different conditions of p63 perturbation and cross reference with other public available data, highlighted a transcriptional network in which p63 acts in combination with its targets to drive and control specification and development of multilayered epithelia.

## Results and Discussion

### The new p63 targets

In our previously published list of 186 p63 targets the percentage of validation was almost complete, suggesting that additional relevant targets were below the stringent threshold. We reanalysed our data with new criteria and extended the list of putative targets to 1259 gene ID: 701 from the CpG island and 579 from the promoter arrays: 21 locations were in common. This low overlap between the two platforms was already observed [10] and reflects the substantial differences of the clones spotted on the two arrays. The complete list of the gene ID is in Table S1, together with genomic coordinates of both the positive CpG island clones and the promoter regions (see materials and methods for additional mapping information). Independent ChIPs from HaCaT cells with two different p63 antibodies, both recognising all p63 isoforms, performed on 40 randomly selected *loci* from this list, indicated *in vivo* binding on 25 (data not shown). This lower validation rate was expected for two reasons: (i) the lower stringency could enhance the noise; (ii) we only used one amplicon per locus, thus probably missing nearby p63 binding sites. A comparison with a genome wide p63 location analysis with a non keratinocyte cell line [9] showed that 188 *loci* -14.8%- are common, supporting the validity of our list. Therefore, we considered the new list worth of further analysis.

**Figure 1:**
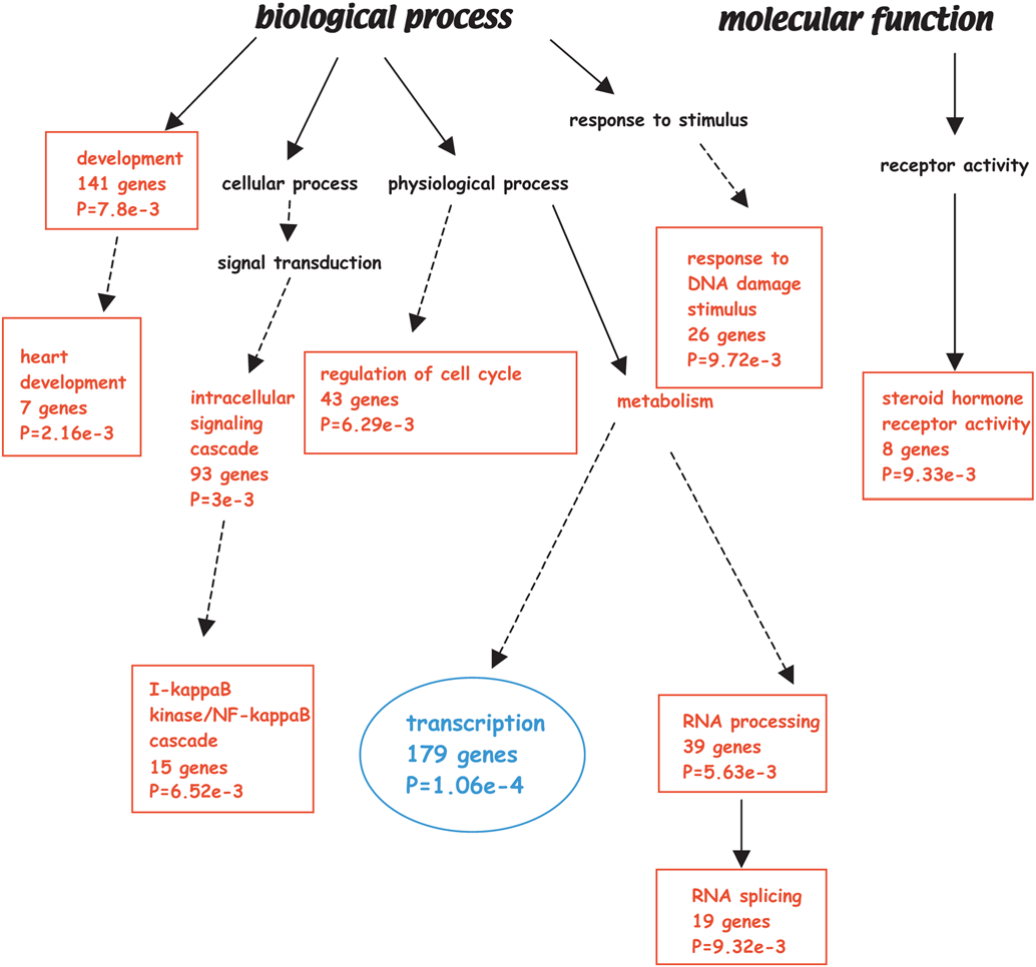
GO enrichment tree of p63 targets. Selected enriched GO categories and their respective number of genes with associated p values are depicted in red. Scheme adapted from Figure S1.

We performed a functional classification through the WebGestalt Gene Set Analysis Toolkit, which allows handling of a large number of genes and performs statistical enrichment of GO terms [13]. The results are visualized as a tree shown in Figure S1 and a more simplified version is in Figure 1. The two major statistically enriched categories were *development* (141 genes) and *transcription* (179 genes) (Figure 1). These two classes emerged also from our previous restricted list [10], reinforcing the idea that a transcriptional cascade is the basic event in developmental programs regulated by p63. RNA processing (39 genes) was found enriched, confirming our previous classification and suggesting an important role of p63 in global translational volume. New categories were also significantly enriched: intracellular signaling cascade, in particular *IkappaBkinase/NF-kappaB cascade*; *regulation of cell cycle*; *response to DNA damage stimulus*; *steroid hormone receptor activity* and, surprisingly, *heart development*. The gene ID of the GO categories mentioned above are listed in Table S2.

Concerning *NFkappaB*, there are several studies implicating this signaling pathway in development and homeostasis of the skin [14] and of skin appendages [15]. Furthermore, IKKa is a transcriptional target of p63 [16], [17] and c-Rel can interact with p63 in squamous carcinoma cells [18]. *Cell cycle* regulation was somewhat expected, given the role of p63 in cancer biology [19]–[23] and maintenance of “stemness” in epithelial biology [24], [25]. In particular the enrichment of the subcategory of *regulation of caspase activity* (Figure S1) corroborates the role of p63 in the apoptotic pathway [26], [27] and confirms a recent finding linking p63 to caspase8 and its regulator cFLIP [28].

*Response to DNA damage stimulus* was not totally unexpected, given the similarity to p53; indeed, an elegant study highlighted the long term function of TAp63 in the female germ line cells [27].

As for *Steroid hormone receptor activity*, the function of VDR and RAR/RXR in skin biology or ER/AR on glandular development is well documented [29], [30]. We were somewhat surprised by the paucity of adhesion/migration categories, since two expression screenings indicated p63 at the center of specific adhesion program [11] and metastatic invasion [12]. This may be due to lack of adequate representation of these particular genes on our arrays, or to a poor annotation of GO for such genes. Finally, since there is only one piece of evidence of p63 expression in the heart [31], it is hard to speculate on its role in this particular organ development, but it may open new windows of investigation.

**Figure 2:**
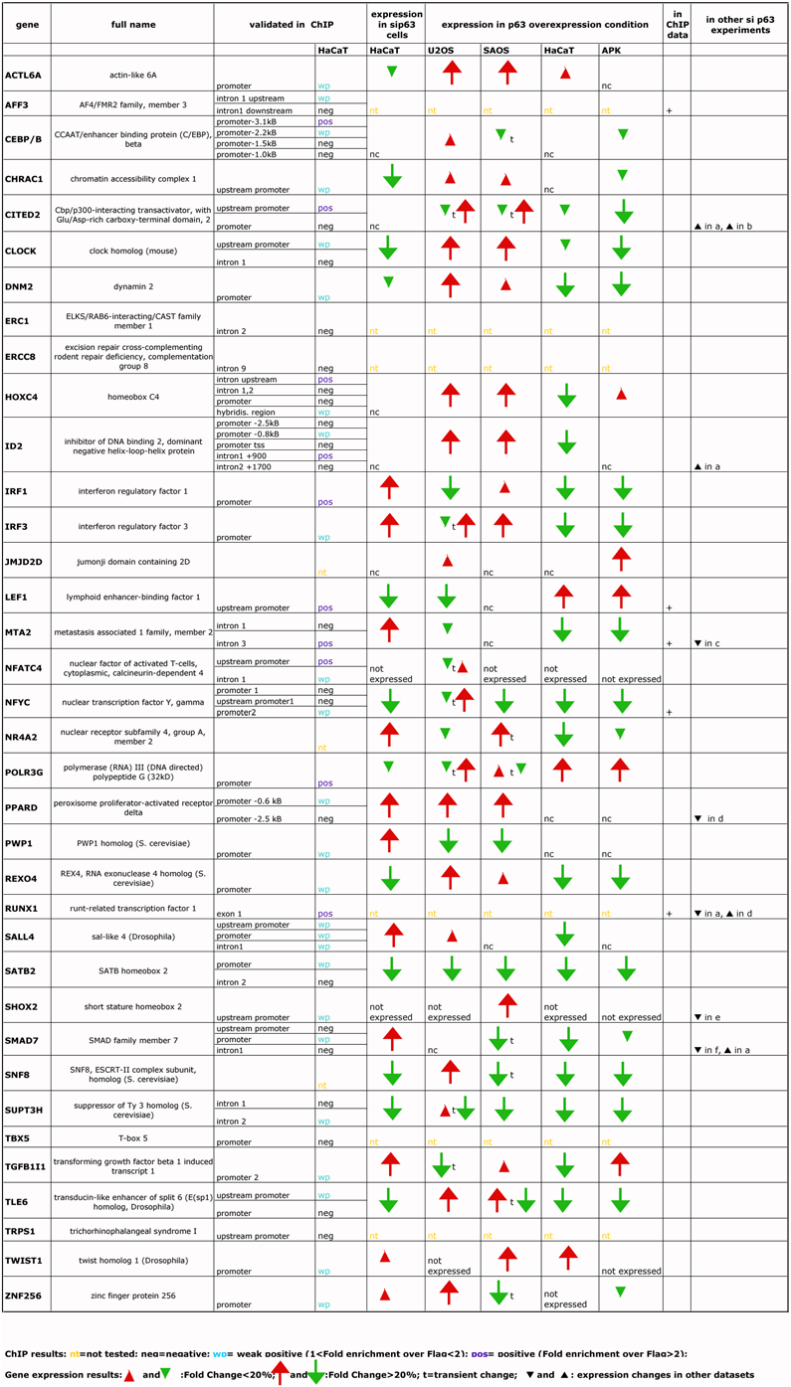
Summary of the validating assays for the 36 transcriptional regulators targeted by p63. The results of the ChIPs on the different selected genomic regions are indicated as positive (pos) when fold enrichment over flag was >2, weak positive (wp) when 1< fold enrichment <2 or negative (neg) if fold enrichment <1. nt  =  not tested. Expression fold changes are indicated with an upward red arrow if the gene was upregulated in the sip63 HaCaT sample or a downward green arrow if the gene was downregulated. The size of the arrow is indicative of the amount of the fold change: thin for changes <20% and thick for changes >20%. nc  =  no change. For the overexpression studies the same representation is depicted with the addition of 2 different time points for the ΔNp63α overexpression in U2OS and the induction of SAOS; the small t indicates a transient change of expression. For HaCaT and APHK only the ΔNp63α overexpression is considered. The + sign in the ChIP data column indicates the presence of the gene in the binding data of [9]. The arrowheads in the other sip63 experiments column indicate expression changes in the cited datasets: a [11], b [33], c [34], d [32], e [12] and f [9].

The *transcription* class was the most significantly enriched (Figure 1) and we decided to deeply analyze this list of 179 genes (Table S3). We selected 36 genes and tested them in three functional assays for their ability to respond to p63: (i) p63 binding *in vivo* in HaCaT cells, in promoter regions and other parts of their locus, guided by an *in silico* analysis for putative p63/p53 binding sites (see material and methods); (ii) endogenous expression after silencing p63 in HaCaT; (iii) expression after ΔNp63α overexpression in 4 cell lines: U2OS, SAOS, HaCaT and adult primary human keratinocytes (APHK). Finally, we monitored the presence of each gene in available datasets generated after p63 silencing in various epithelial cells [9], [11], [12], [32]–[34]. We considered a gene targeted by p63 if it fulfilled two of these three assays. The results are summarized in Figure 2 (Raw data of all the experiments are available upon request; see Figure S2 for controls). Only 5 genes did not meet the set criteria (AFF3, ERC1, ERCC8, TBX5 and TRPS1): therefore, we assumed that the list of 179 genes represents p63 targets with a confidence of 86%.

We observed different scenarios in terms of expression changes: increased expression in sip63 condition and decreased expression in p63 overexpression -IRF1, IRF3, MTA2, NR4A2, PWP1, SMAD7- or the reverse -ACTL6A, LEF1-, suggesting a direct repression or activation, respectively. In other cases, the direction of changes was the same for the two opposite conditions: decreasing -NFYC, SNF8, SATB2, TLE6- or increasing -JMJD2D, PPARD, TWIST1-, indicating that p63 may not be the only factor influencing their transcription rate. Finally, the variable extent of variation for some genes -CEBP/B, CHRAC1, CLOCK, POLR3G, SALL4, TGFB1I1, ZNF256- and the differential direction in the various lines suggest a key role for the cellular context.

**Figure 3:**
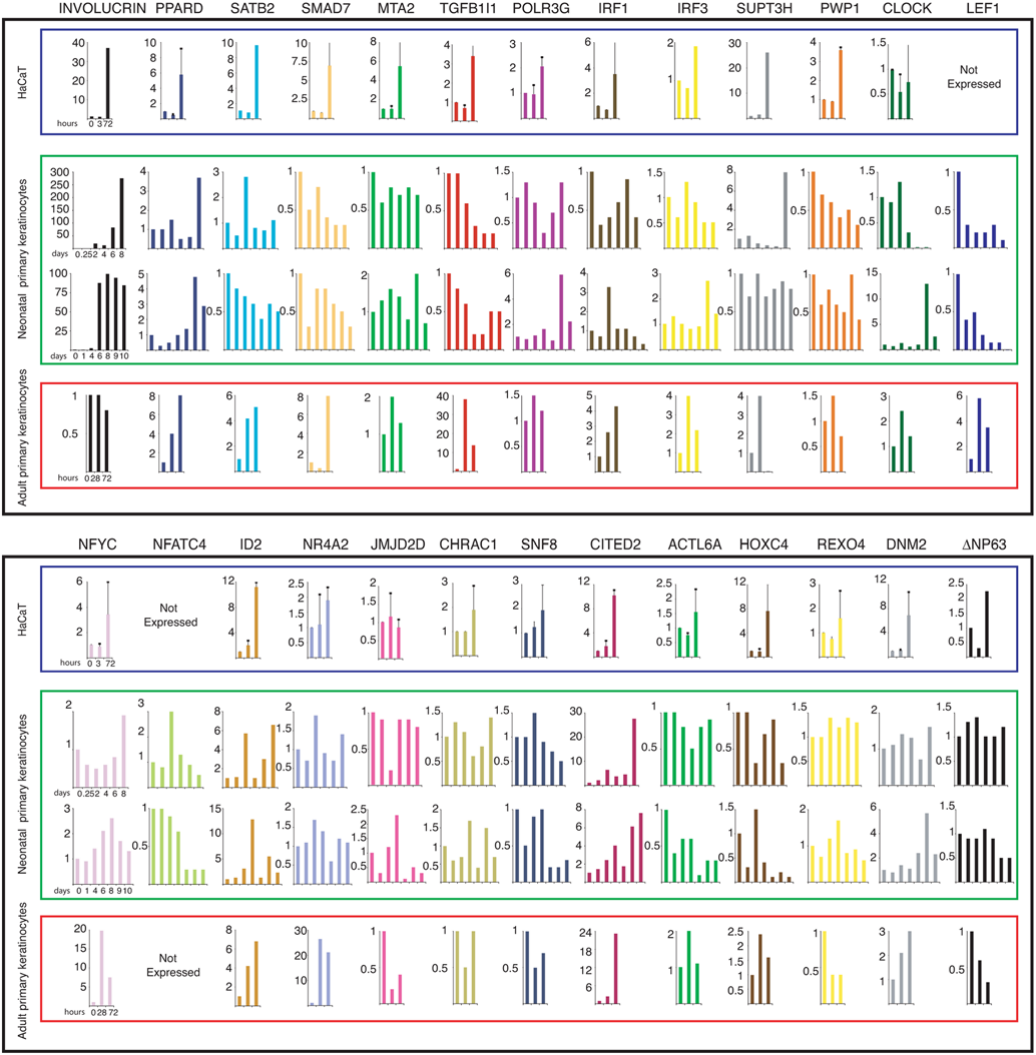
Expression of selected genes in differentiating HaCaT and PHK. Q-PCR analysis of the indicated genes in HaCaT (boxed in blue, mean and SD of two different experiments), neonatal (boxed in green) and adult (boxed in red) human primary keratinocytes after CaCl_2_ differentiation. Time points of RNA collection are indicated on the bottom of the first chart in each row. Bars represent expression fold changes respect to the 0 time point. Please, note the different scale on the y axes of each chart. Data were normalized for GAPDH expression and INVOLUCRIN was chosen to monitor the differentiation status.

To evaluate the role of these targets in keratinocyte biology, we monitored the expression of 26 genes in Q-PCR, at different time points, during differentiation of HaCaT cells, adult and neonatal primary keratinocytes (Figure 3). Expression changes of 24 genes are observed; additional genes and differentiation controls are shown in Figure S3. The general message is clear-cut: all p63 targets involved in transcriptional regulation vary during differentiation, albeit to different extents and in different directions. In particular, most increase in HaCaT and APHK, the exception being CLOCK and JMJD2D in the former, PWP1, JMJD2D, CHRAC1, SNF8 and REXO4 in the latter. In neonatal keratinocytes, the patterns are much more complex: some genes -TGFB1I1, SMAD7, PWP1, LEF1, SNF8, ACTL6A and HOXC4- are dramatically decreased at later time points, while others significantly increase -PPARD, SUPT3H, ID2, CITED2- (Figure 3). This different behavior of neonatal versus adult primary keratinocytes is quite striking and may reflect different skin differentiation program in neonatal and adult life; it may depend on the source of primary keratinocytes: foreskin versus breast, but may also reflect the different differentiation conditions (see materials and methods). Of note, ΔNp63 transcripts are also expressed differently in response to differentiation stimuli in the three systems (Figure 3). In summary, in keeping with the p63-led control in human keratinocytes, the majority, and possibly all transcriptional regulator targets vary in expression during differentiation, suggesting a role in keratinocytes biology.

### A network of Transcriptional targets

The dramatic phenotype observed in p63 null mice points to the essential role of p63 in the initiation and maintenance of the epithelia [7].

**Figure 4:**
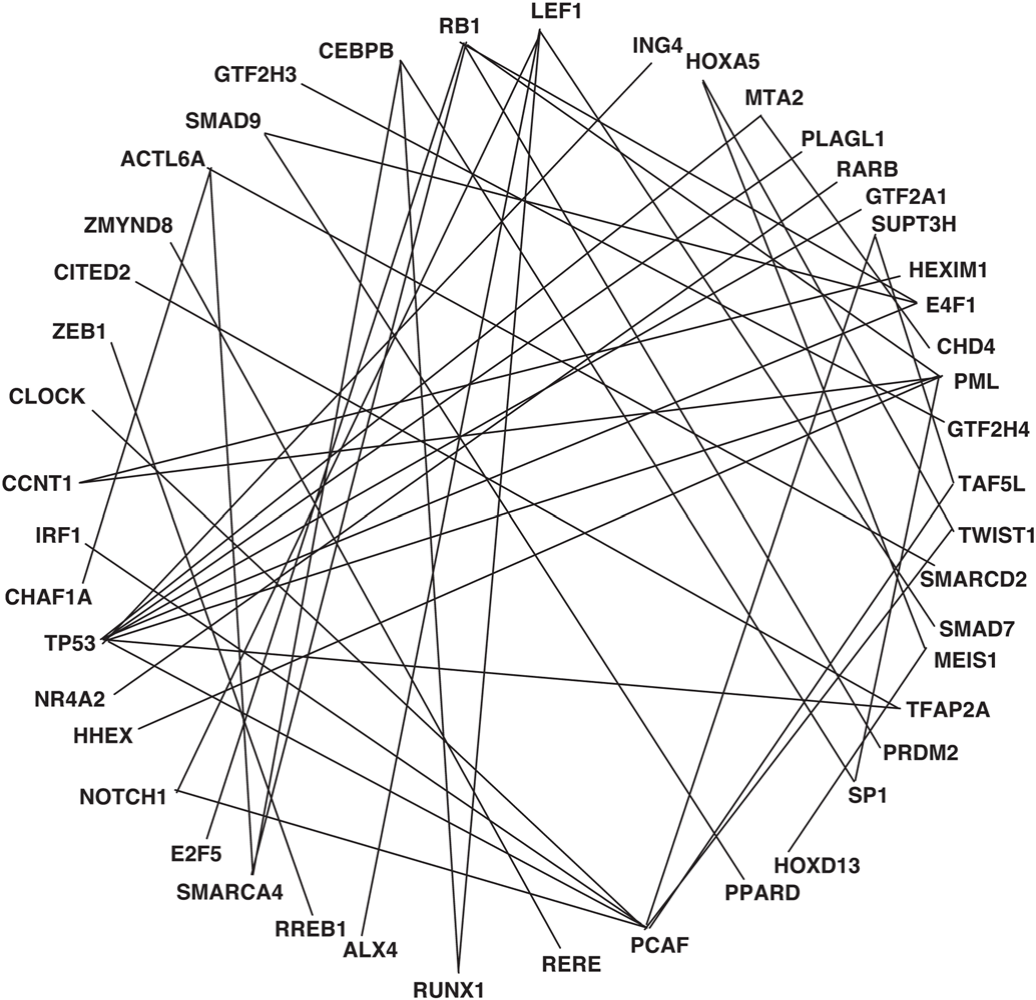
An inner core of p63 transcriptional targets. Schematic representation of the documented interaction of the different p63 transcriptional targets.

Using public available databases of interaction BIOGRID and HPRD (http://www.thebiogrid.org/ and http://www.hprd.org/) we built an “interactome” among the transcriptional regulators of our list for which information was available (Figure 4). It is evident that there are multiple interactors -TP53, PCAF, PML, RB1, LEF1, CEBP/B-, and others with one partner. Notwithstanding the obvious bias related to the enormous amount of information gathered for some of them, there is evidence for a transcriptional network sustaining the different programs in keratinocytes, with many functional interactions contributing and helping p63 to initiate these events.

**Figure 5:**
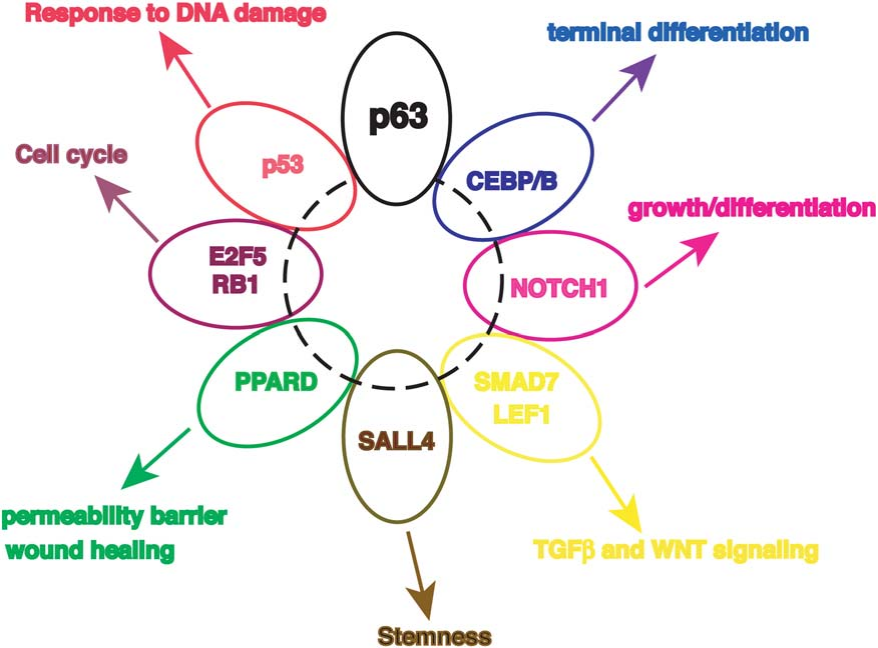
Link of p63 with transcriptional targets. Schematic representation of the recruitment and interplay of p63 and some of its transcriptional targets to direct specific function of the skin.

Elegant studies in the muscle differentiation system identified a handful of transcription factors (TF) -MRFs and MEF2- targeting preferentially a large number of TFs, which, in turn, are contributing to the differentiation process [35]. In the hematopoietic system, very complex cooperative and antagonistic interplays between a considerable number of TFs, as well as timing of their expression, regulate the different lineage specifications from the hematopoietic stem cells [36]–[38]. The epidermis shares features with the above mentioned systems, namely the necessity to produce terminally differentiated cells, but it also has peculiarities. In fact, it is a continuously differentiating organ which not only provides the main barrier of the body to the external environment, but it also needs to respond in a quick way to different types of external *stimuli,* ranging from chemical and physical agents, to microbial attacks, to mechanical stresses and wound healing. A complex relationships among these different programs -self-renewal, differentiation, protection from DNA-damage, immune response, and regeneration- is expected. Therefore, the skin is at the same time simpler than the hematopoietic system, in that essentially only one type of terminally differentiated cell is produced, but still very complex, for the need to deal with the exterior. We provide evidence that the p63 transcriptional network is interconnected with several nodes -or hubs- of single or small groups of transcriptional regulators that are responsible for the different physiologic function. Response to DNA damage is a task that p63 undertakes personally, in conjunction with p53. The CEBPs are important in a number of terminal differentiating systems [39]; E2Fs in promoting cell-cycle progression [40], [41]; NOTCH1 is coupling growth and differentiation [42]; SMAD7 and LEF1 connect to TGFβ and WNT signaling [43]. PPARD is implicated in the homeostasis of the permeability barrier of the skin [44]. Thus, recruitment of different sets of transcriptional regulators by p63 might initiate specific response in the skin (Figure 5).

In summary, rather than a simple hierarchical “pyramid” with p63 on top or a “master-slave” relationship, p63 can be viewed as a connector. This scheme would provide a greater level of plasticity required for the different tasks the epidermis is fulfilling and we expect that the expression of p63 can be modulated by many of these TFs: indeed, we have already started to dissect the reciprocal interplay for one of the regulated TFs: CEBP/D [45].

Finally, we identified SALL4, one of the very upstream TF in the specification of different blastocysts derived stem cell lineages [46] and important in the stem cell network [47]–[49]. Furthermore, human mutation in SALL4 give rise to the Duane-radial ray syndrome, also known as Okihiro syndrome, an autosomal dominant disorder characterized by upper limb anomalies, ocular anomalies, and, in some cases, renal anomalies [50], [51]. Thus, there is considerable overlap with syndromes caused by p63 mutations. The overlap is most pronounced in the limb developmental defects, which likely originate in defective development of the apical ectodermal ridge or maintenance of stem cells of this specialized epithelium. For epithelial development of the skin, SALL4 seems less important as skin defects are not a notable feature of SALL4 mutations. Nevertheless, given the importance of p63 in the stem cells of multilayered epithelia [24], this connection is worth further investigation. Our future goal is to dissect all these interactions, by focusing on the individual hubs of the system, to understand the single contribution of each factor.

## Materials and Methods

### Analysis of binding data and location

The hybridization scheme of the immunoprecipitated material on the 12K CpG island and the 12K promoter arrays was described in [10]. For the CpG island array we considered positive all the clones that satisfied the described criteria: >2 fold enrichment in the α-p63 samples over the negative antibody -α-flag- samples in each of the three performed experiments. For the promoter arrays, we considered all the spots for which more than 70% of the pixels fluoresced with intensity above the median fluorescence intensity of the background plus two standard deviation and those showing enriched IP/control ratio >2 in all four slides were considered positive.

The sequences of the positive CpG islands clones were retrieved as described before [10]. Approximately 2 kb of genomic regions both 5′ and 3′ of the CpG islands clones (or of the transcription start sites for the positive promoter clones) were considered and one or more gene ID were assigned to each clone, if they laid in these regions. The genomic coordinates of the positive CpG islands clones (based on the sequence information retrieved from http://data.microarrays.ca/cpg/) and the −800/+200 regions of the positive promoter clones are supplied in Table S1

GO enrichment analysis was performed at http://bioinfo.vanderbilt.edu/webgestalt with all the 1259 gene id retrieved, but only 1159 were recognized as such. The default settings used were the WebGestalt_Human reference gene set and the Hypergeometric statistical test.

The interaction databases BIOGRID and HPRD can be found at these addresses http://www.thebiogrid.org/ and http://www.hprd.org/

### Analysis of p63/p53 binding site

In order to predict the position of p63 binding sites we used PWM descriptors [52] of p63 and p53 binding preferences. For p63 we used the matrix reported in [53], while for p53 we employed the one found in Jaspar database [54]. Those two profiles were used to score every oligonucleotide (of the same length of the profile) composing the analysed sequences according to the method described in [55]. Oligonucleotides scoring more than 0.80 with at least one of the two matrices were chosen for further investigation. Oligonucleotides scoring more than 0.85 with at least one of the two half site versions of the profiles were also considered.

### Analysis of sip63 microarray data

#### HaCaT [12] and MCF10A [11]

The data were provided by the authors as average of the control (Ctrl) class linear signal and average of the p63 RNA interference (p63si) class linear signal. The Affymetrix probe-sets were ranked according to log2 (p63si/Ctrl), weighted by the log10 (expression signal) in the two classes; probe-sets with a signal smaller than an arbitrary thresholds in Ctrl or p63 were removed from the list of the down-regulated and up-regulated, respectively; the filtering and the weighting were introduced to avoid the selection of probe-sets with small absolute variation but large ratio. Down-regulated (high in Ctrl) and Up-regulated (high in p63si) were selected applying a symmetric threshold. A very similar ratio between up-regulated and down-regulated was obtained even without filtering and weighting, but simply using the log2(ratio).

#### Mouse primary keratinocytes [33]

The data were provided by the authors as raw .CEL files. The data were imported from .CEL files using the Affy package (R/Bioconductor), and the rma statistic was generated for the probe-sets using the rma package (R/Bioconductor). The genes were ranked according to an empirically corrected signal-to-noise: mean.siRNA - mean.Ctrl)/(sd.siRNA+sd.Ctrl+constant). The FDR was estimated as the ratio between the number of probe-sets selected for certain thresholds of the real and the random signal-to-noise; the random signal-to-noise was calculated after the independent random permutation of all the expression matrix rows. The random permutation of the expression matrix columns could not be used due to the limited number of the control class replicates (only two). The constant in the signal-to-noise formula was set to 1, after a few assessments of observed positives and FDR with different values of the constant. The lists of down-regulated probe-sets were generated for 12.2% and 7.6% estimated FDR.

#### HaCaT [34]

Only one replicate was available for each class. Therefore we used the Sscore package (R/Bioconductor) to generate differentiality statistics [56]. The data were imported from .CEL files using the Affy package (R/Bioconductor). The p-values were calculated under the normality assumption for the Sscore distribution, as recommended by the authors. The raw p-values were then corrected using the Benjamini-Hochberg method.

For **ME180** [9] and **human primary keratinocytes** [32] the lists were used as supplied by the authors.

#### Chromatin Immunoprecipitation

The procedure for ChIP was essentially as described previously [10] using 3–5×10^6^ HaCaT cells per 10–15 µg antibody. Two specific α-p63 antibodies were used: a rabbit polyclonal generated in our lab [10] and the mouse monoclonal 4A4 (Santa Cruz), and the negative control one was the α-flag (Sigma). The immunoprecipitated DNA was analyzed with region specific primers both in semi quantitative standard PCR assays and in Q-PCR.

#### Cell culture, transfection, *in vitro* differentiation and RNA extraction

HaCaT, SAOS and U2OS cells were maintained in Dulbecco's modified Eagle's medium supplemented with 10% foetal calf serum, 100 IU penicillin and 100 µg streptomycin per ml. First passage primary adult human keratinocytes (APHK) were derived from healthy individuals and grown on a feeder-layer of lethally irradiated 3T3 cells [10]. Neonatal primary human keratinocytes (NPHK) were purchased from CELLnTEC (Bern, Switzerland) and maintained for 4–5 passages in Progenitor Cell targeted Culture Media (CnT-57, CELLnTEC).

For *in vitro* differentiation assays, HaCaT cells were grown in 0.1% FCS and CaCl_2_ was added to a final concentration of 1.4 mM. Cells were harvested at 0, 3 and 72 hours after calcium induction. APHK were seeded on Mitomycin C (Sigma) arrested 3T3 cells and CaCl_2_ concentration was increased to 1.4 mM. Cells were harvested at 0, 28 and 72 hours after calcium induction. Subconfluent NPHK at passage 5 or 6 were switched to CnT-02 differentiation medium (CELLnTEC, Bern, Switzerland) and 1 day later 1.2 mM CaCl_2_ was added. Cells were harvested at different time points up to 11 days post calcium addition.

Transfections were carried out with Lipofectamine 2000 (Invitrogen, Milan, Italy). 5 µg of ΔNp63α were used for a 100 mm plate of U2OS. 24 µg of TAp63α, ΔNp63α, β and γ were used for 100 mm plates of HaCaT or APHK cells. The p63 expression plasmids were described previously [10]. Induction of SAOS cells (kindly provided by E. Candi) was done with 2 µg/ml of Doxycycline (Sigma-Aldrich, Italy). Cells were collected after 24 h (U2OS, SAOS, HaCaT and APHK) and 48 hours (U2OS and SAOS) post transfection/induction. RNA was extracted with the RNAeasy kit (Qiagen, Milan, Italy) or TriReagent (Sigma-Aldrich, Italy) according to manufacturer's instructions.

For siRNA experiments, 4×10^6^ HaCaT cells were seeded onto 100 mm plates, allowed to attach to the substratum and transfeceted using Lipofectamine 2000 with 60nM siRNA scramble (Ambion/Applied Biosystems, Austin, Texas, USA) or si directed against the DNA binding domain of p63 [34]. After an overnight incubation, a second transfection was performed and cells were collected 48 h later.

1 or 2 µg of RNAs were reverse-transcribed using Superscript II (Invitrogen) and cDNAs were normalized to GAPDH levels.

#### Q-PCR analysis of ChIP and RT

A Biorad MyIQ single colour thermal cycler and a SYBR Green PCR Master mix was used in all Q-PCR experiments. Specificity of products was monitored with a heat dissociation curve. For ChIP, fold enrichment was calculated with the formula 2^−ΔΔCt^ where the Ct represented the threshold cycles of the input, the specific antibody and the negative antibody; a further normalization with the enrichment obtained on a negative genomic region (centromeric satellite 11) was applied. For RT-PCR, fold changes of expression were calculated with the formula 2^−ΔΔCt^ where the Ct represented the threshold cycles of the specific gene and GAPDH. The 0 time point was always taken as the reference point in each set of experiments and the si scramble point was the reference in the si experiment. A list of the ChIP and RT primers used is provided in Table S4.

## Supporting Information

Figure S1 GO tree from Webgestalt
(2.07 MB EPS)

Figure S2 Controls of p63 silencing and overexpression. Western Blot with a-p63 antibody on total extracts of silenced HaCaT (top), transfected U2OS, HaCaT and APHK, and induced SAOS cells, to detect levels of p63 reduction or different p63 isoforms overexpression. In the silencing experiments, a-vinculin was used as a loading control.
(1.27 MB EPS)

Figure S3 Expression analysis of selected genes in differentiating keratinocytes. Standard RT PCR analysis of two additional targets in differentiating NPHK and differentiation control expression of Filaggrin and Keratin5 in APHK. GAPDH was used to normalize the cDNAs
(1.03 MB EPS)

Table S1 Extended list of p63 targets: 1259 gene id and genomic coordinates. The back rows are genes coming from the CpG island array, while the blue ones are from the promoter array. The genes in common between the two platforms are in red. The annotation in the ChIP column is the same as in Table I. s are data from [1]. The letters in the column other sip63 experiments are the same as in Table I. The other worksheet lists the genomic coordinates of the positive clones from both CpG and promoter arrays. They can be uploaded on the UCSC genome browser (human, March 2006 assembly) 1. Birkaya B, Ortt K, Sinha S (2007) Novel in vivo targets of DeltaNp63 in keratinocytes identified by a modified chromatin immunoprecipitation approach. BMC Mol Biol 8: 43
(0.24 MB XLS)

Table S2 Gene ID in the different GO categories
(0.03 MB XLS)

Table S3 179 enriched trascriptional target from the Webgestalt classification. Additional references cited in this table: [2]–[7] 2. Lo Iacono M, Di Costanzo A, Calogero RA, Mansueto G, Saviozzi S, et al. (2006) The Hay Wells syndrome-derived TAp63alphaQ540L mutant has impaired transcriptional and cell growth regulatory activity. Cell Cycle 5: 78–87. 3. Trink B, Osada M, Ratovitski E, Sidransky D (2007) p63 transcriptional regulation of epithelial integrity and cancer. Cell Cycle 6: 240–245. 4. Laurikkala J, Mikkola ML, James M, Tummers M, Mills AA, et al. (2006) p63 regulates multiple signalling pathways required for ectodermal organogenesis and differentiation. Development 133: 1553–1563. 5. Bernassola F, Oberst A, Melino G, Pandolfi PP (2005) The promyelocytic leukaemia protein tumour suppressor functions as a transcriptional regulator of p63. Oncogene 24: 6982–6986. 6. Ortt K, Raveh E, Gat U, Sinha S (2008) A chromatin immunoprecipitation screen in mouse keratinocytes reveals Runx1 as a direct transcriptional target of DeltaNp63. J Cell Biochem 104: 1204–1219. 7. Guttormsen J, Koster MI, Stevens JR, Roop DR, Williams T, et al. (2008) Disruption of epidermal specific gene expression and delayed skin development in AP-2 gamma mutant mice. Dev Biol 317: 187–195.
(0.04 MB XLS)

Table S4 List of primers used in ChIP and RT-PCR experiments.
(0.03 MB XLS)
